# Patients with Advanced Pancreatic Cancer Treated with Mistletoe and Hyperthermia in Addition to Palliative Chemotherapy: A Retrospective Single-Center Analysis

**DOI:** 10.3390/cancers15204929

**Published:** 2023-10-11

**Authors:** Anna Lena Hohneck, Largsi Sadikaj, Lara Heinemann, Maik Schroeder, Hartmut Riess, Annette Gerhards, Iris Burkholder, Stefan Heckel-Reusser, Julia Gottfried, Ralf-Dieter Hofheinz

**Affiliations:** 1 Department of Cardiology, Angiology, Haemostaseology and Medical Intensive Care, University Medical Centre Mannheim, Medical Faculty Mannheim, Heidelberg University, 69117 Heidelberg, Germany; 2 European Center for AngioScience (ECAS), German Center for Cardiovascular Research (DZHK), Partner Site Heidelberg/Mannheim, 68167 Mannheim, Germany; 3 Onkologische Praxis Kaiserslautern, 67655 Kaiserslautern, Germany; 4 Department of Haematology and Oncology, University Medical Centre Mannheim, Medical Faculty Mannheim, Heidelberg University, 69117 Heidelberg, Germanyralf.hofheinz@umm.de (R.-D.H.); 5 Klinik Öschelbronn, 75223 Oeschelbronn, Germany; 6 AnthroMed Öschelbronn, Centrum für Integrative Medizin, 75223 Oeschelbronn, Germany; h.riess@anthromed-oeschelbronn.de (H.R.);; 7 Department of Nursing and Health, University of Applied Sciences of the Saarland, 66117 Saarbruecken, Germany; 8 Heckel Medizintechnik GmbH, 73728 Esslingen, Germany

**Keywords:** integrative oncology, mistletoe therapy, hyperthermia, advanced pancreatic cancer

## Abstract

**Simple Summary:**

Pancreatic cancer is associated with poor survival, despite advances in anti-cancer treatment. Most patients are diagnosed at an advanced stage, so the treatment goal is prolongation of survival and maintenance of quality of life instead of cure. In this context, integrative therapeutic approaches have become increasingly important in recent years. Integrative therapies are usually not used alone but complementary to conventional treatment to optimize its effect. Positive effects have been described for symptom alleviation, e.g., pain and quality of life improvement by the additional use of integrative therapies. However, the available data are not sufficient for a conclusive assessment regarding a possible influence on survival. The present work sought to describe the outcome of patients with advanced or metastatic pancreatic cancer not amenable to curative treatment, who received either conventional therapy or additional complementary integrative mistletoe and/or hyperthermia.

**Abstract:**

This retrospective analysis investigated the influence of integrative therapies in addition to palliative chemotherapy in patients with advanced pancreatic cancer, treated at a single institution specialized in integrative oncology between January 2015 and December 2019. In total, 206 consecutive patients were included in the study, whereof 142 patients (68.9%) received palliative chemotherapy (gemcitabine/nab-paclitaxel 33.8%; FOLFIRINOX 35.9%; gemcitabine 30.3%) while the remainder were treated with best supportive and integrative care. Integrative therapies were used in 117 of 142 patients (82.4%) in addition to conventional chemotherapy, whereby mistletoe was used in 117 patients (82.4%) and hyperthermia in 74 patients (52.1%). A total of 107/142 patients (86.3%) died during the observation period, whereby survival times differed significantly depending on the additional use of integrative mistletoe or hyperthermia: chemotherapy alone 8.6 months (95% CI 4.7–15.4), chemotherapy and only mistletoe therapy 11.2 months (95% CI 7.1–14.2), or a combination of chemotherapy with mistletoe and hyperthermia 18.9 months (95% CI 15.2–24.5). While the survival times observed for patients with advanced pancreatic cancer receiving chemotherapy alone are consistent with pivotal phase-III studies and German registry data, we found significantly improved survival using additional mistletoe and/or hyperthermia.

## 1. Introduction

Pancreatic cancer continues to be associated with poor survival, despite scientific endeavors [1]. The treatment of patients with pancreatic cancer in Germany is based on the updated German S3 guideline from 2021 [2]. FOLFIRINOX or gemcitabine (plus nab-paclitaxel) or vice versa are used as first- or second-line treatment. In addition, stereotactic body radiation therapy has been shown to be an effective option for inoperable locally advanced pancreatic cancer [3]. Compared with conventional radiotherapy, stereotactic body radiotherapy is associated with shorter treatment times and can achieve pain relief in most patients with an acceptable toxicity rate [4,5]. Unfortunately, the treatment goal for most patients is the prolongation of survival and maintenance of quality of life instead of cure [6,7]. In this context, integrative therapeutic approaches have become increasingly important in recent years [8]. While the German S3 guideline “Complementary medicine in the treatment of cancer patients” [9], first published in 2021, addresses the increasing interest of patients and caregivers in integrative therapies, the current version of the S3 guideline on pancreatic cancer makes little reference to this (yet) [2]. Integrative therapies are usually not used alone, but complementary to organ-specific, guideline-based treatment to optimize its effect [10]. Moreover, for patients with pancreatic cancer who cannot tolerate first-line treatment as specified in the guidelines, or who decline to undergo such treatment, best integrative/supportive care may contribute to alleviate tumor symptoms, e.g., pain, and to improve the quality of life [11,12]. In terms of integrative oncology treatment, the most comprehensive data relate to mistletoe therapy as a phytotherapeutic agent [13,14,15,16]. So far, the data available are not sufficient for a conclusive assessment regarding a possible influence on survival [17]. However, there are several data from systematic reviews/meta-analyses and RCTs that support the use of mistletoe to improve quality of life in patients with solid tumors [14,16,18,19], which is why the administration of mistletoe extract for quality of life improvement was included in the guideline for complementary medicine (evidence-based recommendation, level of evidence 1a) [9]. The evidence basis for the use of hyperthermia therapy is smaller; however, data from RCTs and cohort studies indicate that the addition of hyperthermia to standard treatments might be able to increase treatment efficacy in cancer patients [20,21,22].

A key obstacle in the treatment of pancreatic cancer is the immunosuppressive tumor microenvironment, which prevents penetration of chemotherapeutic drugs and anti-tumor immune cells [23,24]. In addition, the dense stromal tissue hampers the delivery of oxygen, causing a hypoxic milieu which aids tumor cells to escape conventional therapies [25]. For this reason, a combined approach using hyperthermia to modify the tumor microenvironment in patients with pancreatic cancer could be useful [26,27]. Hyperthermia has been used in integrative oncology for decades, including whole-body hyperthermia (WBH) and locoregional hyperthermia [28,29,30]. The intention of hyperthermia is generally not to directly destroy cancer cells but to amplify the immune response, which can be achieved via different mechanisms. Moderate whole-body hyperthermia leads to improved blood flow and oxygenation of the surrounding tumor microenvironment, which enhances the effects of chemotherapy [31,32]. In addition, a variety of heat shock proteins are released, which play an active role in antigen recognition in dendritic cells, resulting in a cytotoxic T-cell response [33,34,35]. In general, whole-body hyperthermia uses electromagnetic waves (infrared radiation) to heat the body, while regional procedures involve capacitive fields or microwave antenna systems that lead to selective heating in the tumor [31]. 

The present work sought to describe the experience of patients with advanced or metastatic pancreatic cancer not amenable to curative treatment who were treated at a single institution specialized in integrative oncology. Patients received either conventional therapy or additional complementary integrative mistletoe and/or hyperthermia.

## 2. Materials and Methods

The present retrospective analysis includes *n* = 206 consecutive patients who were treated at the Klinik Öschelbronn between January 2015 and December 2019 due to advanced or metastatic histologically confirmed pancreatic ductal adenocarcinoma. No formal exclusion criteria were in place, and the records and follow-up information of all patients treated within this 5-year period were evaluated.

The study was conducted according to the principles of the Declaration of Helsinki and was approved by the ethical committee of the Landesärztekammer Baden-Württemberg, Stuttgart, Germany (registration number F-2020-160). Data protection was in accordance with the EU Data Protection Directive. 

### 2.1. Baseline Characteristics

Since this is a retrospective evaluation, no systematic information on performance status (ECOG) can be provided. In order to create a relatively comparable cohort, patients who received only the best supportive care were excluded from further analysis, so that the main part of this manuscript refers to *n* = 142 patients with advanced pancreatic cancer who received palliative chemotherapy.

### 2.2. Mistletoe Therapy and Hyperthermia

Both mistletoe and hyperthermia are part of an integrative treatment program that is offered to patients seeking complementary therapy options. Since there is no standard approach to these therapies, treatment was guided by the therapy regimens in the available literature and practical guidelines [36,37,38].

All patients were instructed about the additional use of complementary integrative mistletoe and/or hyperthermia, which is a cost-neutral offer, and gave their informed consent. It should be emphasized that this was only an offer and patients could decide at any time to opt in or out of this additional therapy without giving any reason.

Patients consenting to or requesting mistletoe treatment received subcutaneous injections of Viscum album extract two to three times weekly, according to the treatment regimen in the MISTRAL study [36]. There are mistletoe derivatives from different companies (e.g., Iscador or Abnoba viscum or Helixor), which are interchangeable [17]. These derivatives are registered as phytotherapeutic agents and approved for patients undergoing palliative treatment for cancer. In some patients, treatment with mistletoe derivatives started with a single subcutaneous injection with Abnoba viscum Fraxini at a dose of 20 mg. This type of mistletoe and dose frequently induces fever reactions in patients. Patients undergoing fever-inducing mistletoe treatment afterwards generally receive subcutaneous mistletoe therapy as outlined above. 

Hyperthermia techniques included loco-regional radiofrequency (13.56 MHz) hyperthermia with an Oncotherm EHY2000 device (Oncotherm Kft, Budaörs, Hungary) and moderate WBH with a heckel-HT3000 device (Hydrosun Medizintechnik GmbH, Müllheim, Germany) using water-filtered infrared-A (wIRA) or a heckel-HT2000 device (Heckel medizintechnik GmbH, Esslingen, Germany) using diffuse reflection-scattered infrared A/B. The targeted body core temperature for moderate WBH, also denoted as fever-range hyperthermia, is 38.5–40.5 °C [38]. Patients were warmed up under secure conditions; vital sign tracking (heart rate, oxygen saturation) and continuous rectal temperature measurement were used to monitor core body temperature until the target temperature was achieved, which was then maintained for 60 min. This was followed by a one-hour cooling down period. Sedation is usually not necessary for sessions that do not exceed 180 min. According to the available practical guidelines, WBH is generally administered once or twice a week in direct connection with chemotherapy during an inpatient stay and then repeated according to the chemotherapy regimen or based on subjective tolerance [38]. 

### 2.3. Statistical Analysis

Quantitative parameters were summarized using descriptive statistics (N, arithmetic mean, standard deviation, median, first and third quartile), and qualitative characteristics were evaluated in the form of frequency tables. 

Median overall survival and annual survival rates were calculated as time from date of detection of metastases or local recurrence to death or last contact, according to Kaplan and Meier methodology, and reported along with 95% confidence intervals. Differences in Kaplan–Meier curves between all (sub)groups were tested using the global logrank test.

Survival was analyzed for the following (sub)groups:

Total population of patients with advanced pancreatic cancer;

Subgroup of patients receiving chemotherapy;

Subgroup of patients receiving chemotherapy and complementary integrative mistletoe and/or hyperthermia. 

Due to logistical reasons and availability, as there are only a few institutions in Germany that offer integrative therapies, not all patients started immediately with complementary integrative mistletoe and/or hyperthermia in addition to conventional treatment. In order to avoid the bias that patients who started therapy later had a hypothesized survival advantage because they previously had longer survival, patients were divided into an early versus late complementary treatment group to assess the effect of the time of treatment initiation. Patients who received integrative treatment with mistletoe within two months after the initiation of chemotherapy or three months regarding hyperthermia are referred to as “early group” and the remainder as “late group”.

## 3. Results

**Figure 1:**
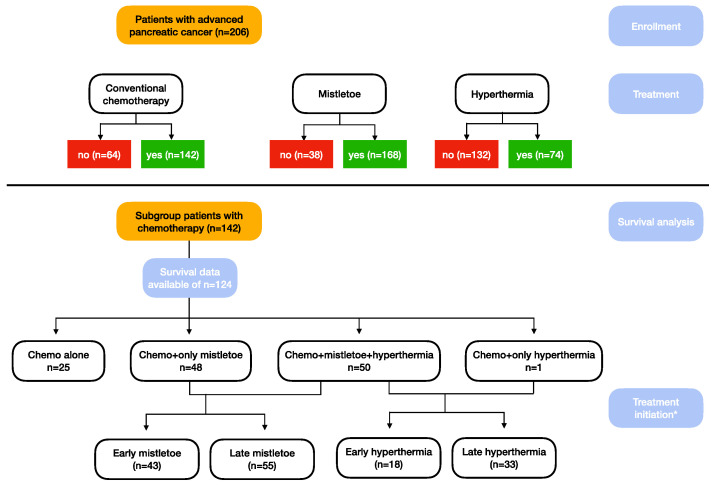
CONSORT diagram * time of treatment initiation (early treatment defined as mistletoe therapy within the first 2 months, hyperthermia within 3 months).

A total of 206 consecutive patients with advanced/metastatic pancreatic adenocarcinoma were included in the current analysis. Of those, 64 patients were unable to receive or refused chemotherapy. These patients are presented only as part of the total population and are excluded from further analyses (CONSORT diagram, Figure 1). In brief, 51 of these patients received mistletoe, 13 of them in combination with hyperthermia.

### 3.1. Baseline Characteristics of Patients Receiving Chemotherapy with or without Mistletoe or Hyperthermia (n = 142)

In total, 77 patients were male (54.2%) and 65 patients (45.8%) were female, with a median age at diagnosis of 66.7 years. 

Overall, 42 patients (29.6%) had undergone a surgical resection of the primary tumor. In 14 (9.6%) patients, the tumor localization was unknown. Local recurrence or unresectable primary tumor was documented in 26 (18.3%) patients. The most common metastatic site was found to be the liver (84 (59.2%)), followed by the peritoneum (56, (39.4%)) and lymph nodes (46 (32.4%)). A total of 32 (22.5%) patients had received adjuvant therapy (Table 1). 

### 3.2. Treatment Regimens

A total of 42 of these 142 patients (30.0%) had prior adjuvant therapy with a curative intent. Subsequently, all 142 patients (100%) received palliative first-line treatment. The median treatment duration was 90 days (53.0;153.0). The most common component of first-line treatment was FOLFIRINOX in 51 patients (35.9%), followed by gemcitabine/nab-paclitaxel in 48 patients (33.8%). Forty-three patients (30.3%) were treated with gemcitabine monotherapy.

A total of 70 patients (49.3%) received second-line treatment. The median treatment duration was 61 days (31.0;96.0). Gemcitabine was used most frequently in 33 patients (23.2%), followed by gemcitabine/nab-paclitaxel in 27 patients (19.0%). FOLFIRINOX was received by 10 patients (7.0%).

A third-line treatment was only used in 17 patients (12.0%). The median treatment duration was 89.5 days (45.5;106.0) (Table 2). 

### 3.3. Integrative Therapies

Integrative therapies were used in 117 of 142 patients (82.4%) in addition to conventional chemotherapy, whereby mistletoe was used in 117 patients (82.4%) and hyperthermia in 74 patients (52.1%). 

The median treatment duration for mistletoe was 41 days (8.0;213.0) and 57 days (1.0;208.0) for hyperthermia, with a median number of four sessions (1.0;6.5). WBH was used in 60 patients (81.1%) and local hyperthermia in 26 patients (35.1%). Of these, treatment was applied to the pancreas/upper abdomen in 22 patients (84.6%) and to unspecified other regions in 8 patients (30.8%). Bones or lymph nodes were not treated in any of the patients and the lungs in only one patient (3.8%) (Table 3). 

### 3.4. Survival Analysis 

Of the 142 patients receiving chemotherapy, survival data were available for 124 patients, of whom 107 (86.3%) had died. Median overall survival was 14.2 months (95% CI 11.5–15.8). Further survival analysis, stratified for treatment with chemotherapy alone, use of chemotherapy and only mistletoe therapy, and chemotherapy in combination with mistletoe and hyperthermia revealed significantly different survival data for both patients receiving additional mistletoe therapy (*p* = 0.02) and the combination of both (*p* < 0.001). Since only one patient received hyperthermia without mistletoe treatment, further analyses for “only hyperthermia” were not conducted.

**Figure 2:**
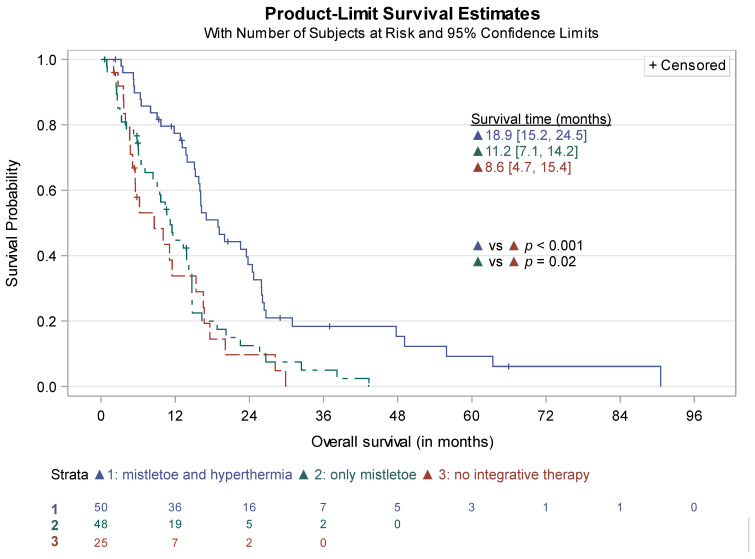
Survival time of patients receiving chemotherapy (*n* = 142).

Median survival for patients receiving chemotherapy alone (*n* = 25) was 8.6 months (95% CI 4.7–15.4), while patients treated with chemotherapy and mistletoe therapy (*n* = 98) survived a median of 11.2 months (95% CI 7.1–14.2). Median survival for patients receiving the combination of chemotherapy with the additional use of mistletoe and hyperthermia (*n* = 51) amounted to 18.9 months (95% CI 15.2–24.5) (Figure 2).

Data on survival times and 1-year, 2-year, and 5-year survival rates are listed in Supplemental Table S1. 

### 3.5. Time of Treatment Initiation

**Figure 3:**
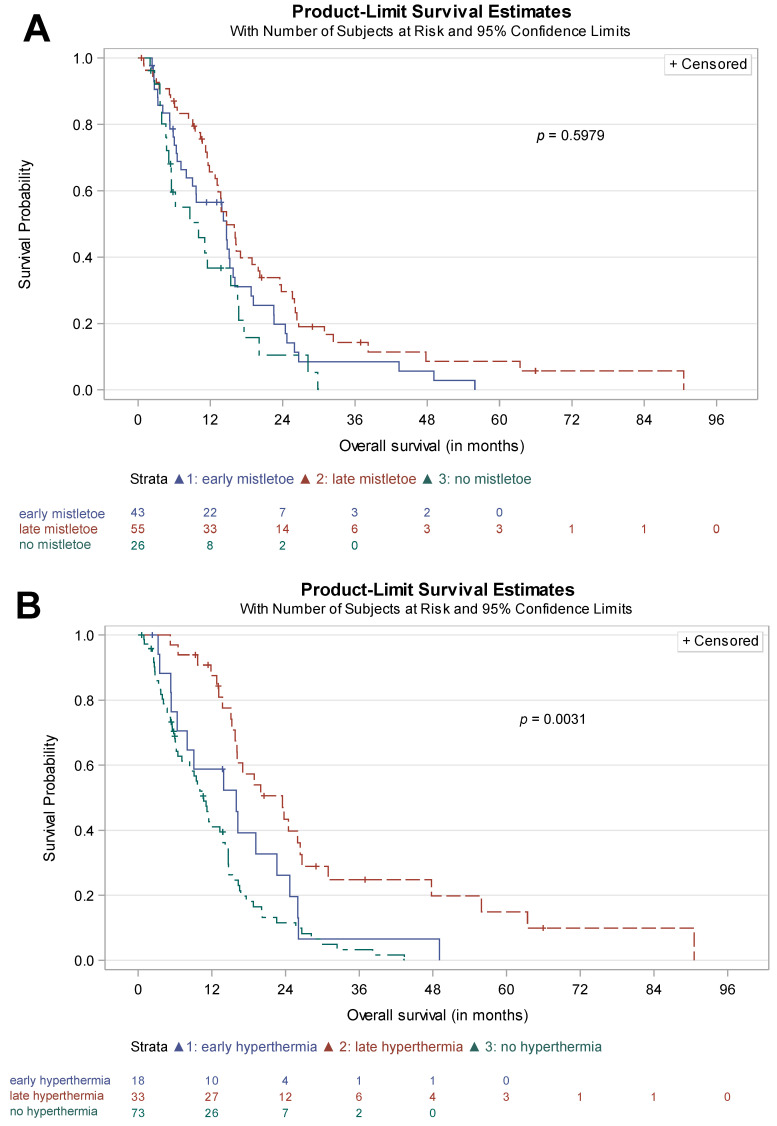
Effect of the time of initiation of the integrative therapy. *P* value reported: global logrank test; (**A**) mistletoe therapy; (**B**) hyperthermia.

As outlined above, patients receiving chemotherapy and complementary integrative mistletoe and/or hyperthermia were allocated to an early versus late complementary treatment group, according to the time of treatment initiation of the integrative therapy. The corresponding Kaplan–Meier curves are shown in Figure 3A mistletoe; Figure 3B hyperthermia.

A significant difference in survival curves was found for hyperthermia therapy (*p* = 0.003), while no statistical difference was observed for mistletoe therapy (*p* = 0.60). 

Median survival for early hyperthermia (*n* = 18) was 16.0 months (95% CI 5.4–22.6), while late hyperthermia (*n* = 33) resulted in 23.5 months (95% CI 15.8–26.4). In the mistletoe groups, median survival was almost identical (early mistletoe (*n* = 43) 14.7 months (95% CI 7.1–15.8) vs. late mistletoe (*n* = 55) 14.7 months (95% CI 12.8–20.0)).

Supplemental Table S2 gives an overview of the survival data dependent of the time of initiation of the integrative therapy.

## 4. Discussion

The present retrospective analysis reports the use of integrative therapeutic approaches in addition to conventional chemotherapy in patients with advanced pancreatic cancer who were treated at a single institution specialized in integrative oncology. Over 80% of the patients of the reported cohort received some type of integrative therapy, which, however, is not the standard in Germany but an offer to the local population covered by the German public health system. In addition, a selection bias must be taken into account since patients that attend a specialized center are by definition accepting of the approach. Compared to patients undergoing chemotherapy alone, encouraging results indicating favorable survival for patients receiving a combination of chemotherapy and complementary mistletoe and/or hyperthermia were observed. This effect was more pronounced for the combination of mistletoe and hyperthermia than for mistletoe alone. Interestingly, there was a significant difference in survival with regard to initiation of the integrative therapy, which will be discussed in detail below.

Integrative therapies are used in addition to conventional chemotherapy in the treatment of pancreatic cancer or as part of best integrative/supportive care in a small proportion of patients [8]. In the patient cohort reported herein, mistletoe therapy was used frequently (in 82% of the total population), while hyperthermia therapy was used in 36% of patients. A large number of preclinical and clinical studies as well as various reviews and meta-analyses are available on the use of mistletoe therapy in tumor diseases, which show an increase in quality of life, [14] while data in terms of a possible prolongation of survival time are less conclusive [13]. Favorable results of mistletoe treatment could also be shown in pancreatic cancer patients [39,40]. The interpretation of the vast literature on mistletoe, however, is complicated by the fact that mistletoe extracts from different manufacturers are not directly comparable as the pharmaceutical processes are used for extraction so, consequently, the composition differs. This could be one of the reasons for the divergent results of the different studies. Patients in our cohort received different mistletoe extracts, with Iscador being fermented, while Abnoba viscum is an aqueous extraction of a pressed juice, and Helixor is obtained as a non-fermented fresh juice. 

Currently, there are no comprehensive clinical trials investigating the benefit of hyperthermia in patients with pancreatic cancer. Bull et al. reported good responses in 37 chemotherapy-resistant patients treated with cisplatin, gemcitabine, and daily interferon-alpha combined with moderate, long-duration (6 h) WBH, achieving a 43% overall response rate and, in addition, an improvement in quality of life. Interestingly, in the subgroup of pancreatic cancer, 5 of 7 patients achieved a partial remission [21]. In patients with resected pancreatic cancer, the addition of regional hyperthermia and cisplatin to an adjuvant gemcitabine-based treatment failed to improve disease-free survival (the primary endpoint of this trial). However, a trend in improved post-progression and overall survival in the experimental arm was seen [41]. It must be noted that the treatment regimens in the discussed studies were outside the guideline recommendations, which is why these results are not generalizable. Therefore, based on the current data, no clear recommendation for the use of hyperthermia can be made.

The data presented here pertain to patients who were treated according to the then-current standards between 2015 and 2019. During this period, chemotherapy for the treatment of pancreatic cancer evolved, and there are new first-line treatment options available, such as the NAPOLI regimen [42]. The median survival of patients who received chemotherapy alone in our cohort was 8.6 months, which is comparable to data from pivotal trials that compared different therapy regimens such as FOLFIRINOX or gemcitabine/nab-paclitaxel with gemcitabine monotherapy and NALIRIFOX with gemcitabine/nab-paclitaxel (cf. Table 4) or large registries [42,43,44,45]. Moreover, the results of the German tumor registry pancreatic cancer reporting on real world data [46] are also in line with the survival data observed in our cohort. Of course, our data do not have the same power, if only due to the retrospective nature of the study and the limited sample size, which is why the comparison is by no means from a statistical perspective.

With regard to integrative therapies, we are unable to draw direct comparisons between our study population and other cohorts, as previous studies do not provide precise information on this or have only assessed the survival of patients with advanced pancreatic cancer treated with mistletoe or no antitumor therapy [40,47]. The ongoing MISTRAL study investigating the efficacy of mistletoe extract added to a standard treatment in advanced pancreatic cancer will help to determine the impact of mistletoe derivatives on survival in this patient group [36]. 

While the overall survival data in patients receiving mistletoe and hyperthermia are promising, the results of the survival analysis in terms of initiation of the integrative therapy are, at first glance, surprising. While no difference was found in the early and late mistletoe groups, survival was significantly better in the late hyperthermia group. These results, however, should be interpreted with caution because of a potential bias. It is conceivable that the late hyperthermia group comprised more patients who primarily responded to conventional chemotherapy and were only converted to a combination of chemotherapy and integrative therapy approach during the course of treatment. Since there are still no uniform criteria or standardized therapy protocols for the use of hyperthermia, it is difficult to compare the currently available studies. Furthermore, there are different types of hyperthermia: locoregional hyperthermia, WBH, and intraoperative hyperthermia. Both locoregional hyperthermia and WBH were also used in our cohort. In addition, patients in the current analysis were frequently treated with a combination of mistletoe and hyperthermia. 

Due to the poor prognosis of pancreatic cancer and significant side effects associated with first-line therapy, some patients deliberately decide against conventional chemotherapy, weighing the benefits and negative effects. For this reason, it is of utmost importance to highlight therapy alternatives that are tolerable and represent a serious alternative to best supportive care. The large number of patients in our cohort who received integrative mistletoe treatment reflects the increasing interest of patients in such a therapy. In the context of integrative therapy approaches, many patients have the feeling that they can actively contribute to their recovery in a way that may go beyond standard therapy. The extent to which this positive mindset could also influence survival has not been sufficiently investigated. 

### Strengths and Limitations

The cohort described herein represents comparably promising survival data from consecutive patients treated at a specialized integrative oncology center. However, this only applies to a tiny proportion of patients in Germany. In addition, a selection bias must be taken into account, as patients who attend a specialized center are by definition accepting of the approach. That these data are building some evidence is supported by the survival times of patients who received chemotherapy alone. Their survival times compare adequately with the data from pivotal studies and registries. A shortcoming of this study is the retrospective study design, which allows only limited conclusions to be drawn. In addition, patients did not receive homogeneous treatment in terms of mistletoe derivatives. Hyperthermia was usually used in combination with mistletoe therapy, so we are not able to estimate the benefit of additional hyperthermia without mistletoe treatment. Due to the observational nature of the study, we did not perform a competing risk analysis.

## 5. Conclusions

Despite advances in oncology research, pancreatic cancer is still associated with poor outcome. For this reason, there is growing interest in the use of integrative therapies from both practitioners and patients, which is also reflected in the implementation of guidelines. Even though pure survival time is not necessarily related to quality of life, this metric is a surrogate for treatment success and is equally an important question that diagnosed patients ask themselves. The described cohort presents survival data from consecutive patients treated at a specialized integrative oncology center. While survival times observed for patients with advanced pancreatic cancer receiving chemotherapy alone are consistent with pivotal phase-III studies and German registry data, we found significantly improved survival using additional mistletoe and/or hyperthermia.

## Figures and Tables

**Table 1:** Baseline characteristics.

		Patients Receiving Chemotherapy (*n* = 142)
Sex male, *n* (%)		77 (54.2)
Age at diagnosis, median (Q1;Q3)		66.7 (59.6;73.0)
Surgical treatment, *n* (%)		42 (29.6)
Tumor localization, *n* (%)		
	Pancreas head	76 (53.5)
	Pancreas body	31 (21.8)
	Pancreas tail	39 (27.5)
	Unknown	14 (9.9)
Local recurrence, *n* (%)		35 (24.7)
Liver metastases, *n* (%)		84 (59.2)
Peritoneal metastases, *n* (%)		56 (39.4)
Lymph node metastases, *n* (%)		46 (32.4)
Lung metastases, *n* (%)		41 (28.9)
Others, *n* (%)		34 (23.9)
Adjuvant therapy, *n* (%)		32 (22.5)

Data are shown as numbers (percentage) or median (Q1;Q3). *n*, number.

**Table 2:** Treatment regimens of *n* = 142 patients with advanced pancreatic cancer.

Treatment Regimens		Patients Receiving Chemotherapy (*n* = 142)
First line treatment, *n* (%)		142 (100)
	Duration of therapy (days), median (Q1;Q3)	90.0 (53.0;153.0)
	Discontinued, *n* (%)	140 (98.6)
	Gemcitabine, *n* (%)	43 (30.3)
	Gemcitabine/nab-Paclitaxel, *n* (%)	48 (33.8)
	FOLFIRINOX, *n* (%)	51 (35.9)
Second line treatment, *n* (%)		70 (49.3)
	Duration of therapy (days), median (Q1;Q3)	61.0 (31.0;96.0)
	Discontinued, *n* (%)	69 (48.6)
	Gemcitabine, *n* (%)	33 (23.2)
	Gemcitabine/nab-Paclitaxel, *n* (%)	27 (19.0)
	FOLFIRINOX, *n* (%)	10 (7.0)
Third line treatment, *n* (%)		17 (12.0)
	Duration of therapy (days), median (Q1;Q3)	89.5 (45.5;106.0)
	Discontinued, *n* (%)	17 (12.0)

Data are shown as numbers (percentage) or median (Q1;Q3). FOLFIRINOX, folinic acid, 5-fluorouracil, irinotecan, and oxaliplatin; *n*, number.

**Table 3:** Use of integrative therapies (mistletoe therapy and hyperthermia) in *n* = 142 patients with advanced pancreatic cancer receiving chemotherapy.

Integrative Therapies			Patients Receiving Chemotherapy (*n* = 142)
(A) Mistletoe therapy, *n* (%)			117 (82.4)
	Treatment initiation after first diagnosis (months), median (Q1;Q3)		4.0 (1.5;12.3)
	Treatment initiation after diagnosis of metastatic spread (months), median (Q1;Q3)		1.9 (0.5;6.1)
	Duration of therapy (days), median (Q1;Q3)		41.0 (8.0;213.0)
(B) Hyperthermia therapy, *n* (%)			74 (52.1)
	Treatment initiation after first diagnosis (months), median (Q1;Q3)		8.9 (3.9;13.8)
	Treatment initiation after diagnosis of metastatic spread (months), median (Q1;Q3)		5.7 (2.2;10.9)
	Duration of therapy (days), median (Q1;Q3)		57.0 (1.0;208.0)
	Number of treatment sessions, median (Q1;Q3)		4.0 (1.0;6.5)
	Whole body hyperthermia, *n* (%)		60 (81.1)
	Local hyperthermia, *n* (%)		26 (35.1)
		Pancreas/epigastrium, *n* (%)	22 (84.6)
		Lung, *n* (%)	1 (3.8)
		Others, *n* (%)	8 (30.8)

Data are shown as numbers (percentage) or median (Q1;Q3). *n*, number.

**Table 4:** Cross trial comparison.

		Median Survival (Months)	95% CI
Conroy et al. (2011) [43], multicenter, randomized, phase 2–3 trial comparing FOLFIRINOX to gemcitabine monotherapy		11.1	9.0 to 13.1
Von Hoff et al. (2013) [44], multicenter, open-label, randomized, phase 3 study comparing gemcitabine/nab-paclitaxel to gemcitabine monotherapy		8.5	7.9 to 9.5
Hegewisch-Becker et al. (2019) [46], prospective clinical cohort study using different regimens (German TPK)		9.2	8.5 to 10.0
Klein-Brill et al. (2022) [45], retrospective cohort study		9.3	8.7 to 9.8
Wainberg et al. (2023) [42], multicenter, open-label, randomized, phase 3 study comparing NALIRIFOX to gemcitabine/nab-paclitaxel		11.1	10.0 to 12.1
Current data			
	Chemotherapy alone	8.6	4.7 to 15.4
	Chemotherapy + only mistletoe	11.2	7.1 to 14.2
	Chemotherapy + combination mistletoe/hyperthermia	18.9	15.2 to 24.5

CI, confidence interval; TPK, Tumour Registry Pancreatic Cancer.

## Data Availability

Data will be made available from the corresponding author upon reasonable request.

## Supplementary Materials

The following supporting information can be downloaded at: https://www.mdpi.com/article/10.3390/cancers15204929/s1, Table S1: Survival of *n* = 142 patients with advanced pancreatic cancer receiving chemotherapy in dependence of an additional integrative therapeutic approach. Table S2: Survival (patients with advanced pancreatic cancer receiving chemotherapy) in dependence of the time of initiation of the integrative therapy (early vs. late after diagnosis).
