# Supplementary material for: Patients with Advanced Pancreatic Cancer Treated with Mistletoe and Hyperthermia in Addition to Palliative Chemotherapy: A Retrospective Single-Center Analysis
Source: Cancers (Basel). 2023 Oct 11;15(20):4929. doi: 10.3390/cancers15204929 (PMC10605673; doi:10.3390/cancers15204929)
Supplement: Supplementary file 1 [file cancers-15-04929-s001.zip › cancers-2571042-supplementary.pdf]

Supplemental Table S1: Survival of n = 142 patients with advanced pancreatic cancer receiving chemotherapy in dependence of an additional integrative therapeutic approach

	Median survival (months)	*1-year survival rate (%)*	*2-year survival rate (%)*	*5-year survival rate (%)*
**Mistletoe and hyperthermia**	18.9<br>[15.2; 24.5]	77.4<br>[62.9; 86.8]	37.3<br>[23.5; 51.0]	9.2<br>[2.6; 21.0]
**Only mistletoe**	11.2<br>[7.1; 14.2]	44.7<br>[29.9; 58.5]	12.5<br>[4.6; 24.4]	0<br>--
**No mistletoe, no hyperthermia**	8.6<br>[4.7; 15.4]	33.8<br>[15.4; 53.2]	9.7<br>[1.7; 26.3]	0<br>--

Survival times are given as months (median (range)) or percentage.

Supplemental Table S2: Survival (patients with advanced pancreatic cancer receiving chemotherapy) in dependence of the time of initiation of the integrative therapy (early vs late after diagnosis)

		Median survival (months)	*1-year survival rate (%)*	*2-year survival rate (%)*	*5-year survival rate (%)*
**Mistletoe therapy**	**early**	14.7<br>[7.1; 15.8]	56.5<br>[40.1; 70.0]	19.8<br>[8.8; 33.9]	0<br>--
	**late**	14.7<br>[12.8; 20.0]	65.7<br>[51.1; 76.8]	29.6<br>[17.7; 42.4	8.66<br>[2.5; 19.5]
**Hyperthermia**	**early**	16.0<br>[5.4; 22.6]	58.8<br>[32.5; 77.8]	26.1<br>[8.3; 48.6]	0<br>--
	**late**	23.5<br>[15.8; 26.4]	87.6<br>[70.2; 95.2]	43.4<br>[25.4; 60.1]	14.9<br>[4.2; 31.7]

Survival times are given as months (median (range)) or percentage.
